# The Impact of Media, Phylogenetic Classification, and *E. coli* Pathotypes on Biofilm Formation in Extraintestinal and Commensal *E. coli* From Humans and Animals

**DOI:** 10.3389/fmicb.2018.00902

**Published:** 2018-05-08

**Authors:** Daniel W. Nielsen, James S. Klimavicz, Tia Cavender, Yvonne Wannemuehler, Nicolle L. Barbieri, Lisa K. Nolan, Catherine M. Logue

**Affiliations:** ^1^Department of Veterinary Microbiology and Preventive Medicine, College of Veterinary Medicine, Iowa State University, Ames, IA, United States; ^2^Interdepartmental Toxicology Program, Iowa State University, Ames, IA, United States; ^3^Department of Population Health, College of Veterinary Medicine, University of Georgia, Athens, GA, United States; ^4^Department of Infectious Diseases, College of Veterinary Medicine, University of Georgia, Athens, GA, United States

**Keywords:** phylogenetic groups, biofilms, *E. coli*, extraintestinal, ExPEC, clustering

## Abstract

Extraintestinal pathogenic *Escherichia coli* (ExPEC) include avian pathogenic *E. coli* (APEC), neonatal meningitis *E. coli* (NMEC), and uropathogenic *E. coli* (UPEC) and are responsible for significant animal and human morbidity and mortality. This study sought to investigate if biofilm formation by ExPEC likely contributes to these losses since biofilms are associated with recurrent urinary tract infections, antibiotic resistance, and bacterial exchange of genetic material. Therefore, the goal of this study was to examine differences in biofilm formation among a collection of ExPEC and to ascertain if there is a relationship between their ability to produce biofilms and their assignment to phylogenetic groups in three media types – M63, diluted TSB, and BHI. Our results suggest that ExPEC produce relatively different levels of biofilm formation in the media tested as APEC (70.4%, *p* = 0.0064) and NMEC (84.4%, *p* = 0.0093) isolates were poor biofilm formers in minimal medium M63 while UPEC isolates produced significantly higher ODs under nutrient-limited conditions with 25% of strains producing strong biofilms in diluted TSB (*p* = 0.0204). Additionally, *E. coli* phylogenetic assignment using Clermont’s original and revised typing scheme demonstrated significant differences among the phylogenetic groups in the different media. When the original phylogenetic group isolates previously typed as group D were phylogenetically typed under the revised scheme and examined, they showed substantial variation in their ability to form biofilms, which may explain the significant values of revised phylogenetic groups E and F in M63 (*p* = 0.0291, *p* = 0.0024). Our data indicates that biofilm formation is correlated with phylogenetic classification and subpathotype or commensal grouping of *E. coli* strains.

## Introduction

Extraintestinal pathogenic *Escherichia coli* (ExPEC) is a pathotype of *E. coli* responsible for morbidity and mortality in a wide range of hosts. In humans, neonatal meningitis *E. coli* (NMEC) is responsible for approximately 28–29% of cases of neonatal bacterial meningitis with a mortality rate of 12% (Gaschignard et al., 2011; Stoll et al., 2011). Uropathogenic *E. coli* (UPEC) is responsible for the greatest number of both catheter-associated urinary tract infections and uncomplicated urinary tract infections worldwide (Flores-Mireles et al., 2015). Serious cases of urinary tract infections can result in pyelonephritis, potentially leading to sepsis and death. Avian pathogenic *E. coli* (APEC) is a non-human subpathotype of ExPEC and the causative agent of colibacillosis in poultry. Collectively, human infections caused by ExPEC strains result in billions of dollars in healthcare costs annually, while APEC also poses a significant financial burden to the poultry industry (Nolan et al., 2013; Flores-Mireles et al., 2015). In addition, ExPEC subpathotypes from different host sources have also been shown to exhibit genomic (Johnson et al., 2007) and phenotypic similarities (Rodriguez-Siek et al., 2005a; Tivendale et al., 2010).

Biofilms are complex communities of surface-associated microorganisms that are enclosed in a structured, highly hydrated extracellular polysaccharide (Donlan and Costerton, 2002; Flemming and Wingender, 2010). In themselves, biofilms are a distinct bacterial lifestyle since biofilm formation promotes resistance to antibiotics and sanitation as well as exchange of DNA (Davey and O’toole, 2000; Beloin et al., 2008). Biofilms can lead to persistent and chronic infections in humans, and an estimated 65% of hospital infections are of biofilm origin (Potera, 1999). Additionally, persistence of APEC in the poultry production environment may be due to biofilm development on plastic surfaces such as water feeding systems (Lindsay et al., 1996; Amaral, 2004). *E. coli* K1 biofilms have also been isolated from neonatal nasogastric feeding tubes (Alkeskas et al., 2015), and biofilm-like intracellular bacterial communities (IBCs) are known to play an important role in the pathogenesis of UPEC since IBC formation allows UPEC to continue colonization of the bladder and resist expulsion (Schwartz et al., 2011; Hannan et al., 2012; Flores-Mireles et al., 2015). The ability of ExPEC to exchange DNA in biofilms is also of concern due to potential acquisition of virulence and antimicrobial resistance plasmids (Johnson et al., 2005; Rodriguez-Siek et al., 2005b).

Although biofilm analyses have been done independently in APEC (Skyberg et al., 2007), UPEC (Soto et al., 2007), and NMEC (Wijetunge et al., 2015), cross comparisons among these ExPEC subpathotypes are problematic due to procedural differences in the studies. This lack of comparative data can be a roadblock to the study of the zoonotic potential of ExPEC, the development of universal mitigation strategies to control ExPEC-caused diseases, and our understanding of ExPEC’s environmental persistence, transmission, and pathogenesis. Additionally, failure to understand the differences in biofilm production between ExPEC and their commensal *E. coli* counterparts, including avian fecal *E. coli* (AFEC) and human fecal *E. coli* (HFEC), can undermine our understanding of ExPEC’s disease pathogenesis and prevent clarity on the importance of biofilm production in the survival of ExPEC outside vs. inside the host.

Another issue that may limit our clarity on the importance of biofilm production to ExPEC pathogenesis is that previous studies (Skyberg et al., 2007; Soto et al., 2007; Wijetunge et al., 2015) of ExPEC biofilm production were analyzed by phylogenetic assignment according to the original typing scheme of Clermont (Clermont et al., 2000). Here, we compare different ExPEC, assigned to different phylogenetic groups using Clermont’s more recent and refined typing scheme (Clermont et al., 2013). Such data may lend great insight into the role of biofilm in pathogenesis since phylogenetic group assignment may predict an *E. coli*’s capacity to cause disease (Picard et al., 1999).

In addition to looking for differences in biofilm production between ExPEC and *E. coli* commensals, among members of different ExPEC subpathotypes, and members of different phylogenetic groups, we sought to determine if these differences were best discerned under three different conditions of growth, including nutrient-poor, nutrient-limited, or nutrient-rich conditions (Skyberg et al., 2007).

In the present study, we seek to remedy deficits in our understanding of ExPEC biofilm production. To do so, biofilm production of ExPEC from APEC, NMEC, and UPEC subpathotypes and their commensal counterparts, all assigned to phylogenetic groups according to Clermont’s original and revised typing schemes and using the same methodology under three conditions of growth was assessed.

## Materials and Methods

### Strain Selection

To assess biofilm formation, 175 *E. coli* isolates were tested including 108 ExPEC strains and 67 commensal *E. coli* strains. These isolates were randomly selected from collections that have been described previously (Rodriguez-Siek et al., 2005a,b; Johnson et al., 2008, 2009; Logue et al., 2017), and efforts were made to ensure that members of all phylogenetic groups were represented. Since Logue et al. (2017) demonstrated natural differences in the distribution of the phylogenetic groups among isolates of the different subpathotypes, this study did not equilibrate the isolates selected based on their subpathotypes or commensal classification, thus avoiding production of erroneous results when generalizing the groups. **Table 1** provides information about the isolates examined in this study, and the data for each strain is found in the Supplementary Table 3.

**Table 1:** Isolates used in this study as classified by subpathotype or commensal category and Clermont’s phylogenetic typing schemes.

	Original scheme						Revised scheme						
		*n*	A	B1	B2	D	A	B1	B2	C	D	E	F
**ExPEC**	APEC	44	11	4	7	22	4	4	6	8	5	9	8
	NMEC	32	8	2	15	7	5	2	15	3	1	1	5
	UPEC	32	9	5	7	11	4	5	5	5	7	1	5
**Commensal**	AFEC	33	9	6	8	10	5	5	4	4	5	5	5
	HFEC	34	9	6	9	10	7	6	8	1	6	1	5
**Sum**		175	46	23	46	60	25	22	38	21	24	17	28


*Extraintestinal pathogenic *E. coli* (ExPEC) subpathotypes examined included avian pathogenic *E. coli* (APEC), neonatal meningitis *E. coli* (NMEC), and uropathogenic *E. coli* (UPEC). Commensal *E. coli* isolates included could be separated by host – avian fecal *E. coli* (AFEC) and human fecal *E. coli*(HFEC).*

### Biofilm Assay

Isolates to be tested were struck from frozen stock to tryptic soy agar (TSA, Difco, BD, Franklin Lakes, NJ, United States) and incubated at 37°C for 18–24 h. Three colonies were selected per strain and incubated for 16 h in Luria Bertani (LB) Miller broth (Difco). These cultures were tested based on methods previously described (O’Toole et al., 1999; Stepanović et al., 2004; Skyberg et al., 2007). Previous work has demonstrated that the assay used here, a 96-well microtiter plate with crystal violet staining, has a high repeatability and broad applicability and is useful for the examination of the early stages of biofilm development (O’Toole et al., 1999; Merritt et al., 2005; Peeters et al., 2008; O’Toole, 2011). After incubation for 16 h, the strains were diluted 1:100 with each broth; these included brain heart infusion (BHI) broth (Bacto), 1/20 diluted tryptic soy broth (TSB; Bacto), and M63 minimal media (Sturgill et al., 2004; Skyberg et al., 2007). 200 μL aliquots of each diluted strain in medium were dispensed into seven wells of a Sarstedt 96-well flat bottom microtest plate (Sarstedt, Nümbrecht, Germany). Uninoculated medium was used as a negative control in the eighth well. Plates were incubated at 37°C for 24 h without shaking before the contents of the plates were decanted, and the plates washed once with sterile deionized water. Following washing, microplates were stained for 30 min with 200 μL of 0.1% crystal violet solution (J.T. Baker Chemical Co., Phillipsburg, NJ, United States). After staining, the plates were washed four times with sterile deionized water and allowed to air-dry for 1 h. Following drying, the biofilms were resolubilized with 200 μL of an 80:20 solution of ethanol and acetone, and 150 μL of the resulting solution was transferred into a new plate. Biofilm formation was quantified by measuring the optical density (OD) of the dissolved crystal violet at 600 nm using an ELx808 Ultra MicroPlate Reader with Gen5 Microplate Reader and Imaging software (Bio-Tek Instruments, Winooski, VT, United States). The ODs of the three biological replicates in seven wells were averaged for each test strain in each medium tested.

### Clermont’s Phylogenetic Typing

All isolates were assigned to phylogenetic groups based on the original and revised Clermont phylogenetic typing schemes (Clermont et al., 2000, 2013). These protocols have been described previously and are based on amplification of the *chuA* and *yjaA* genes as well as the TSPE4.C2 DNA fragment (Clermont et al., 2000) or revised *chuA*, *yjaA*, and TspE4.C2 DNA fragment along with the *arpA* and *trpA* genes (Clermont et al., 2013; Logue et al., 2017). Amplified products were run on a 2% agarose gel in 1× TAE buffer with known controls (ECOR collection) and classification of the strains was determined by the presence or absence of gene products.

### Biostatistics

The ODs measured for the negative control wells for each test strain and medium combination were measured and averaged. The ODs for each test strain in a given medium were averaged and normalized against the negative control by subtracting the average OD of the negative control from the average OD of the test strain. Statistical analyses were performed in MATLAB (Mathworks^®^) using the normalized average data for each test strain in each of the three media. As the obtained ODs deviate from a normal distribution, the Kruskal–Wallis (KW) test (one-way ANOVA on ranks) was used as a non-parametric test to determine significant differences in biofilm formation between the original or revised Clermont’s phylogenetic types or subpathotype or commensal *E. coli* (Kruskal and Wallis, 1952). Multiple comparisons were performed in relation to ANOVA using Tukey’s honest significant difference test (HSD) to reduce the incidence of Type I error (Tukey, 1949). Direct comparisons between two groups were made using the Mann–Whitney U test (MW; Mann and Whitney, 1947).

To reduce the human bias introduced by setting cutoffs for levels of biofilm formation, cluster analysis was performed by an unsupervised machine learning technique. For each medium, isolates were clustered by level of biofilm formation using *k*-means clustering (MacKay, 2003) with four means identified to represent negligible, low, moderate, and high levels of biofilm formation; the use of these four categories is consistent with previous work (Stepanović et al., 2004; Skyberg et al., 2007). The *k*-means algorithm was performed with the L1-norm; 10,000 replicates for each media were performed to produce optimal clustering. When biofilm production was treated as a discrete classifier, the chi-square test of homogeneity was used to determine statistically significant differences. Significance for all statistical tests was determined at the α = 0.05 level.

## Results

### Overall Associations of Medium and Biofilm Production

In the three different media tested, biofilm formation exhibited limited correlation between media (Pearson’s correlation coefficient: M63 to TSB, *r* = 0. 3461, M63 to BHI, *r* = 0. 3217, BHI to TSB, *r* = 0.1892), which suggests that biofilm formation in each medium should be examined individually. In general, the optical densities were significantly greater for isolates when grown in M63 than in diluted TSB or BHI (KW, *p* = 1.492E-09). Supplementary Figure 1 shows the optical densities of each medium for all strains examined with the standard error of mean included.

**FIGURE 1:**
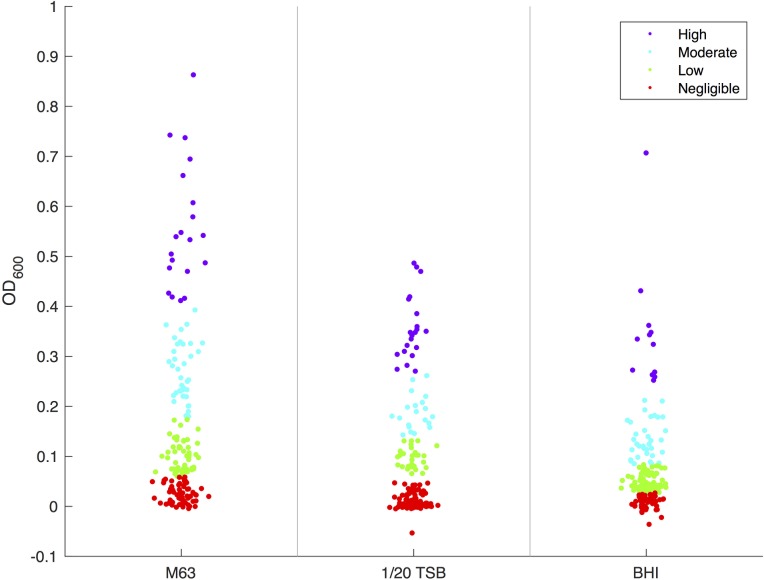
*k*-means Clustering of *E. coli* by media. *k*-means clustering was used to determine the level of biofilm production for each *E. coli* isolate in four categories: negligible (red), low (green), moderate (blue), and high (purple).

When all *E. coli* were classified as a negligible, low, moderate, or high biofilm in each medium using *k-*means clustering (**Figure 1**), approximately 33.1% of *E. coli* strains formed moderate or high biofilms in M63; classification of moderate or high biofilms in diluted TSB and BHI were lower at 23.4 and 26.9%, respectively (Supplementary Table 1). The final OD measurements of each strain tested are available in Supplementary Table 3.

### Associations Between Biofilm Formation by *E. coli* Commensals and ExPEC Subpathotypes

**FIGURE 2:**
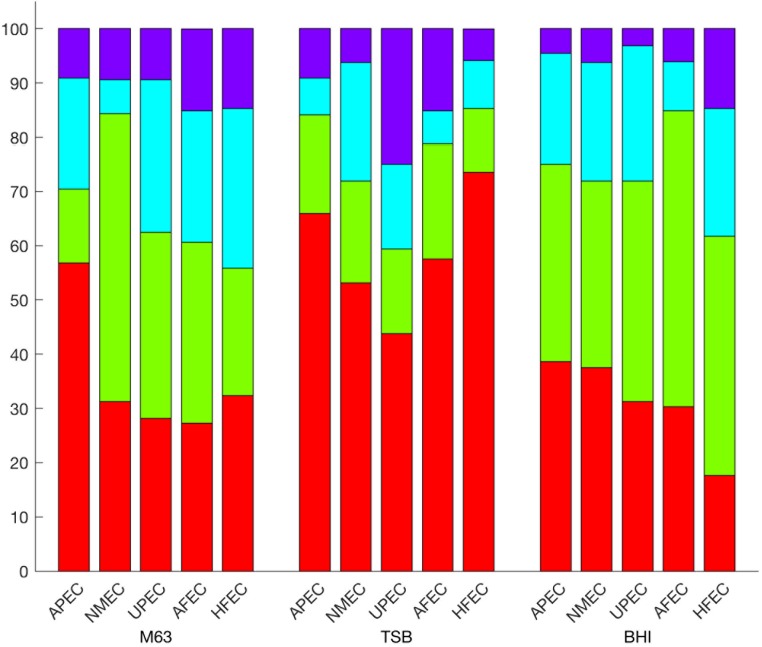
Prevalence of biofilm formation in ExPEC subpathotypes and *E. coli* commensals. Percentage of negligible (red), low (green), moderate (blue), and high (purple) biofilm forming isolates by subpathotype or commensal *E. coli* designation. APEC and NMEC are significantly different in their abilities to form biofilms in M63 (*p* = 0.0064 and 0.0093, respectively).

When all test strains were examined in M63 broth and classified as negligible, low, moderate, or high biofilm producers, strains classified as APEC and NMEC were found to be significantly weaker biofilm producers (χ^2^, *p* = 0.0064 and 0.0092, respectively) among all of the *E. coli* examined. In APEC, the majority of strains (56.8%) produced negligible biofilms while the majority of NMEC (53.1%) produced low-level biofilms (**Figure 2**). UPEC, AFEC, and HFEC strains could not be differentiated from any other *E. coli* groups (MW, *p* = 0.6007, 0.6371, 0.4956, respectively). In addition, the majority of UPEC (62.5%), AFEC (57.6%), and HFEC (52.9%) isolates produced low or moderate biofilms using *k*-means clustering (**Figure 2**).

**FIGURE 3:**
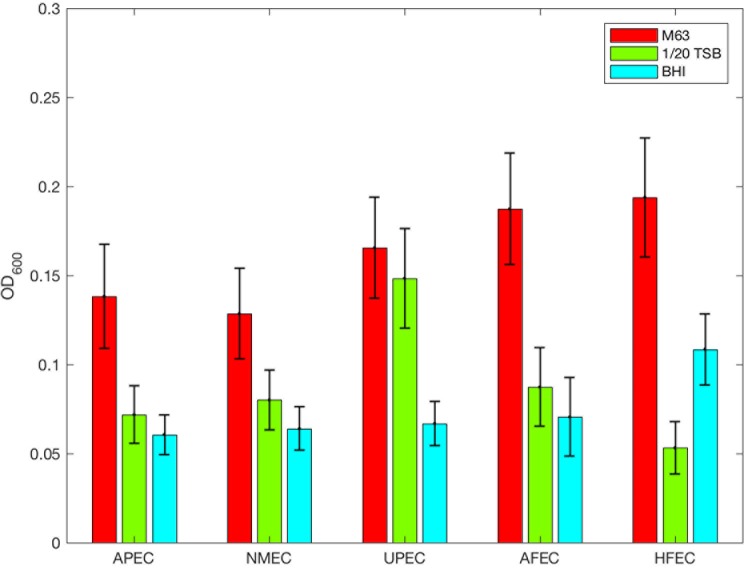
Optical density of ExPEC and commensal *E. coli* strains. Biofilm formation relative to the ExPEC commensal and subpathotype classification and media: M63 (red), 1/20 TSB (green), and BHI (blue). The mean OD_600_ is plotted; error bars represent ± standard error of mean.

In diluted TSB, UPEC strains produced significantly higher ODs compared to APEC, NMEC, AFEC, and HFEC isolates (MW, *p* = 0.02045, **Figure 3**). Even though UPEC did not produce a significant *p*-value (χ^2^, *p* = 0.0505) when the biofilm levels of UPEC strains were categorized, a greater proportion of UPEC strains classified as high-level biofilm formers (25%) compared to any other commensal or ExPEC group (**Figure 2**). In contrast, most (range 53–73%) of the APEC, NMEC, AFEC, and HFEC isolates produced negligible biofilms in 1/20 TSB (**Figure 2**).

In BHI, no significant differences were observed for the ODs of the groups (**Figure 2**), yet more APEC and NMEC strains were categorized as negligible biofilm producers than any other category of biofilm (38.6 and 37.5%, respectively, **Figure 2**). AFEC and HFEC isolates produced more low-level biofilms than any other category of biofilm formation (54.5 and 44.1%, respectively). Additionally, HFEC produced more high-level biofilms than the isolates from any other subpathotype or commensal group (14.7%, **Figure 2**).

### Associations Between Biofilm Formation by *E. coli* and Host

Since APEC and NMEC showed significant OD differences in M63 broth, we attempted to determine if there were differences between *E. coli* isolated from chickens (APEC and AFEC) and humans (NMEC, UPEC, HFEC). Interestingly, no significant differences were observed between strains isolated from the two hosts, suggesting that biofilm formation is not associated solely with the host origin of the strain (Supplementary Figure 2).

### Associations Between Clermont’s Original Phylogenetic Groups and Biofilm Formation

**FIGURE 4:**
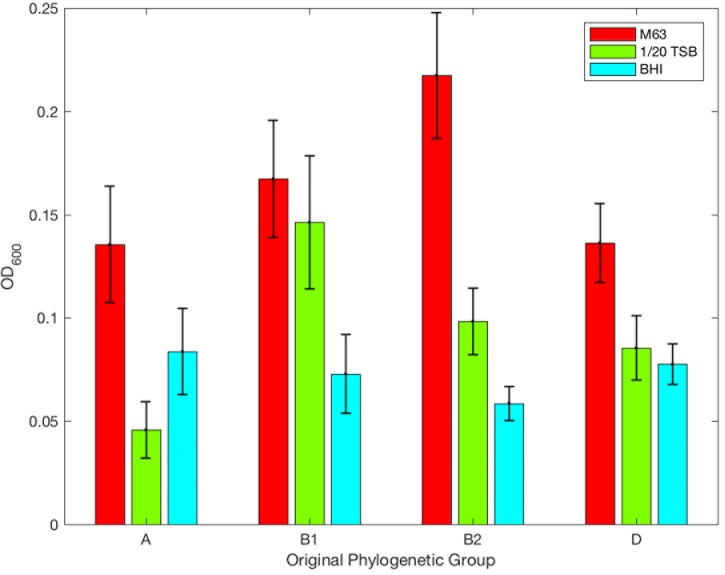
Optical density of *E. coli* isolates as designated using Clermont’s original phylogenetic groups. Biofilm formation relative to Clermont’s original phylogenetic typing scheme and media: M63 (red), 1/20 TSB (green), and BHI (blue). The mean OD_600_ is plotted; error bars represent ± standard error of mean.

Significant differences were observed between the ODs of each isolate classified by Clermont’s original phylogenetic groups when all *E. coli* were compared in M63 broth (KW, *p* = 0.0055, **Figure 4**). The OD of phylogenetic group B2 showed significantly greater biofilm formation than phylogenetic group A (HSD, *p* = 0.0131, **Figure 4**). The majority of phylogenetic group A strains were found to be significantly different (χ^2^, *p* = 0.0172) than the other *E. coli* when the level of biofilm production was examined. A majority of group A isolates produced negligible biofilms (55.6%, **Table 2**). There were no significant differences between groups B2 and B1 or D since a greater prevalence of these isolates produced moderate or high-level biofilms at 43.5, 41.3, and 27.9%, respectively among the isolates examined (**Table 2**).

**Table 2:** Chi-square discrete categorization of *E. coli* by Clermont’s original phylogenetic groups^∗^.

	Negligible	Low	Moderate	High	*p*
**M63**				
A	55.6%	17.8%	15.6%	11.1%	**0.0172**
B1	21.7%	34.8%	39.1%	4.3%	0.0847
B2	21.7%	37.0%	23.9%	17.4%	0.0803
D	39.3%	32.8%	18.0%	9.8%	0.7572
**1/20 TSB**				
A	75.6%	13.3%	6.7%	4.4%	0.0644
B1	47.8%	8.7%	17.4%	26.1%	0.0750
B2	45.7%	23.9%	21.7%	8.7%	**0.0183**
D	62.3%	18.0%	4.9%	14.8%	0.2369
**BHI**				
A	46.7%	28.9%	11.1%	13.3%	**0.0047**
B1	34.8%	34.8%	21.7%	8.7%	0.9032
B2	21.7%	60.9%	13.0%	4.3%	**0.0240**
D	26.2%	39.3%	31.1%	3.3%	**0.0368**

*^∗^Percentages may not add up to 100.0% due to rounding. Values in bold are significant to α < 0.05.*

Like M63, significant differences were observed between all *E. coli* grown in diluted TSB (KW, *p* = 2.999E-3, **Figure 4**). When the ODs of phylogenetic groups B1 and B2 were examined, these groups demonstrated significantly greater biofilm formation than phylogenetic group A (HSD, *p* = 0.01084 and *p* = 8.366E-3, respectively). When the biofilm level of phylogenetic group B2 was compared to others, it was significantly different (χ^2^, *p* = 0.0183) than the other phylogenetic groups, and 45.7% of group B2 produced low or moderate biofilms (**Table 2**). 43.5% of phylogenetic group B1 isolates produced moderate or high biofilms while the majority of phylogenetic group A and D isolates produced negligible biofilms (75.6 and 62.3%, respectively, **Table 2**).

Phylogenetic groups A, B2, and D were statistically different from the other phylogenetic groups (χ^2^, *p* = 0.0047, *p* = 0.0240, *p* = 0.0367, respectively) in BHI when their biofilm levels were assessed. The majority of phylogenetic group A isolates (75.6%) produced negligible or low-level biofilms (**Table 2**). Phylogenetic group B2 isolates (60.9%) formed more low-level biofilms than any other group. Phylogenetic group D strains (31.1%) produced more moderate level biofilms than phylogenetic groups A, B1, or B2 (**Table 2**).

### Associations Between Clermont’s Revised Phylogentic Groups and *E. coli* Biofilm Formation

**FIGURE 5:**
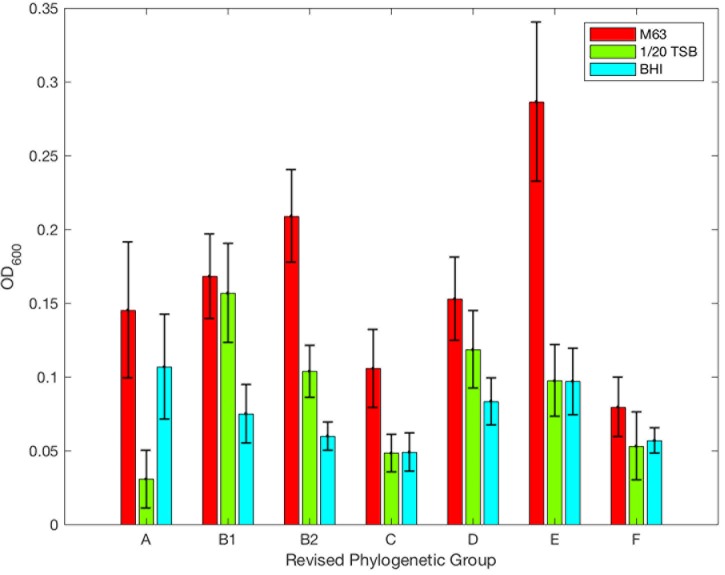
Optical density of *E. coli* isolates as designated using Clermont’s revised phylogenetic groups. Biofilm formation relative to Clermont’s revised phylogenetic group and media: M63 (red), 1/20 TSB (green), and BHI (blue). The mean OD_600_ is plotted; error bars represent ± standard error of mean.

**FIGURE 6:**
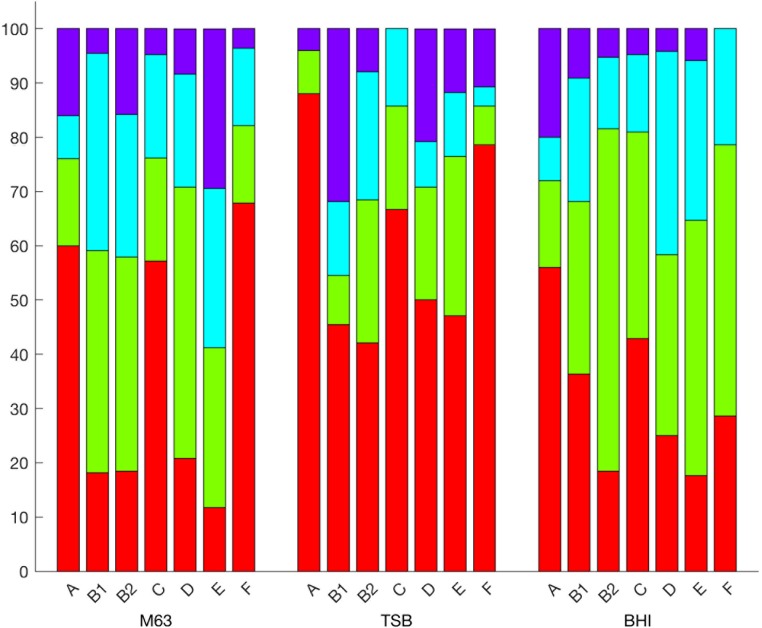
Prevalence of biofilm formation by *E. coli* classified using Clermont’s revised phylogenetic groups in each medium. Cumulative percentage of negligible (red), low (green), moderate (blue), and high (purple) biofilm forming isolates by Clermont’s revised phylogenetic groups. The significant differences between groups are shown in Supplementary Table 2.

A Kruskal–Wallis test based on the ODs of each isolate classified by Clermont’s revised phylogenetic typing scheme identified significant differences among the revised phylogenetic groups and strains grown in M63 (*p* = 1.627E-5). The OD of phylogenetic groups A and F were less than groups B2 or E (*p* values in Supplementary Table 3, **Figure 5**). The ODs of phylogenetic group C compared to any different phylogenetic group were not significant, but the difference between group C and E was suggestive of a possible difference (*p* = 0.0527). When their biofilm levels were compared to the remaining isolates using the χ^2^ test, phylogenetic groups A, E, and F were significantly different (*p* = 0.0243, *p* = 0.0290, *p* = 0.0024, respectively, Supplementary Table 2). In M63, phylogenetic groups A and F overwhelmingly produced negligible biofilms, (60 and 67.9%, respectively, **Figure 6**). In contrast, phylogenetic group E was the most prolific biofilm former with 58.8% of isolates forming moderate or high biofilms (**Figure 6**). Of the other phylogenetic groups, 50% of phylogenetic group D isolates formed low biofilms, and 57.1% of phylogenetic group C isolates formed negligible biofilms (**Figure 6**). The majority of phylogenetic group B2 isolates (65.8%) produced low or moderate biofilms (**Figure 6**).

Like M63, a Kruskal-Wallis test based on the OD of tested isolates and Clermont’s revised phylogenetic typing scheme identified significant differences in diluted TSB (*p* = 4.501E-7, **Figure 6**). The ODs of the different phylogenetic groups revealed phylogenetic groups A and F were characterized by significantly lower biofilm ODs than other phylogenetic groups with higher optical densities observed for B1, B2, D, and E (HSD, *p*-values in Supplementary Table 3, **Figure 6**). Phylogenetic group C was not statistically different from any group. When the level of biofilm formation was determined via *k*-means clustering, phylogenetic groups A, B1, and B2 were all significantly different from the other phylogenetic groups (χ^2^, *p* = 0.0161, *p* = 0.0176, *p* = 0.0078, **Figure 6**, Supplementary Table 2). A majority of isolates classified as phylogenetic groups A, C, D, and F produced negligible biofilms (88.0, 66.7, 50, and 78.6% respectively, **Figure 6**). A total of 50.0 and 41.2% of isolates from phylogenetic groups B2 and E produced low and moderate biofilms, respectively (**Figure 6**). Isolates identified as phylogenetic group B1 produced more high-level biofilms than any other group in diluted TSB (31.8% of isolates examined, **Figure 6**).

In BHI, phylogenetic groups A and B2 were statistically different from groups other than themselves (χ^2^, *p* = 0.0001 and *p* = 0.0258, Supplementary Table 2). For phylogenetic group A, 56% of isolates produced negligible biofilms while 63.2% of phylogenetic group B2 isolates produced low-level biofilms (**Figure 6**). For phylogenetic group E, 47.1% of the isolates examined formed low-level biofilms (9.1%, **Figure 6**).

## Discussion

In ExPEC, there is substantial morbidity and mortality associated with urinary tract infections and neonatal meningitis in humans as well as colibacillosis in avian hosts. The suffering and costs associated with diseases caused by ExPEC are undoubtedly significant (Nolan et al., 2013; Flores-Mireles et al., 2015). Therefore, we assessed the associations between APEC, NMEC, UPEC, AFEC, and HFEC with biofilm formation using the crystal violet phenotype assay. Additionally, we examined the genetic similarity of the bacterial isolates tested and their ability to form biofilms using Clermont’s original and revised phylogenetic typing schemes. Although previous work has been done surveying biofilm formation in APEC and AFEC (Skyberg et al., 2007), NMEC and HFEC (Wijetunge et al., 2015), UPEC (Soto et al., 2007), and *E. coli* derived from raw meat and eggs (Mellata et al., 2017), this study is the first to examine biofilm formation across the subpathotype and commensal groups under standardized conditions, and the work presented demonstrates a novel, unbiased way to classify biofilms in a high throughput manner.

In order to classify biofilm formation, we utilized a machine learning-based clustering methodology to remove human bias in classifying the negligible, low, moderate, and high biofilm forming groups. The four biofilm categories used in this study were selected to allow for comparison with the earlier work of our lab reported by Skyberg et al. (2007). We believe this method to be advantageous to other methods previously used to distinguish the relative formation of biofilms as we do not classify biofilms by an author drawn cutoff or use a standard deviation system from the control wells (O’Toole et al., 1999; Skyberg et al., 2007; Soto et al., 2007; Mitchell et al., 2015; Wijetunge et al., 2015; Mellata et al., 2017), yet we do note that the approach presented may classify isolates slightly differently than previous methods. In a recent study by Mitchell et al. (2015), 93% of ExPEC-like *E. coli* were found to produce biofilms in Luria–Bertani medium. These numbers differ significantly from our strain classifications here, but these *E. coli* strains were assayed in round-bottom 96-well plates. Additionally, the *E. coli* strains were reported to be isolated from meat and eggs, not ill avian or human hosts (Mitchell et al., 2015). These isolates had their subpathotype identified based on the presence or absence of genes associated with virulence or phenotypic differences. The current study classified the pathotype based on the source of the original isolate and the presence or absence of disease, similar to literature precedent (Skyberg et al., 2007; Soto et al., 2007; Logue et al., 2012). Therefore, APEC isolates were isolated from diseased birds while NMEC and UPEC were isolated from cases of neonatal meningitis or urinary tract infections in humans. AFEC and HFEC isolates were obtained from avian or human hosts, respectively, which did not demonstrate apparent disease.

Interestingly, when we tested ExPEC isolates, we found our results to be analogous to previous work with ExPEC *sensu stricto*. For NMEC, it has previously been reported that approximately 77.4% of isolates form biofilms in Luria-Bertani broth, and 52.8% of isolates form biofilms in a minimal medium (Wijetunge et al., 2015). Although we did not test the nutrient-rich Luria-Bertani medium, we did find that a majority (68.8%) of NMEC formed biofilms in a minimal medium, yet the majority of these biofilms were classified as low-level biofilms (53.1%). Likewise, Skyberg et al. (2007) found that the majority of APEC (range 53.3–98.1%) and AFEC (range 56.3–83.4%) formed “none/weak” biofilms in M63, 1/20 TSB, and BHI. We concur that a majority of APEC (range 70.4–84.1%) and AFEC (range 60.6–84.8%) produce negligible and low biofilms in all media tested, but we found that the production of moderate- or high-level biofilms was slightly greater when compared with Skyberg et al. (2007).

Like APEC and NMEC, subtle differences were observed for UPEC strains when they were examined based on their biofilm classification and previously reported studies. Soto et al. (2007) found 40–43% of UPEC produce biofilms while the current study found that 37.5% of UPEC produced moderate- or high-level biofilms in minimal medium. Nevertheless, the similarity of biofilm production among these ExPEC, even with slightly different methods of biofilm classifications between studies, gives us confidence in utilizing a *k*-means clustering methodology for biofilm classification.

To our knowledge, this is the first time an unbiased cluster analysis-based approach has been used to classify biofilm formation, yet machine learning clustering techniques have been used to examine other biological systems such as sensing contamination in the food industry (Haugen and Kvaal, 1998; Ghasemi-Varnamkhasti et al., 2009) and identification of bacteria responsible for urinary tract infections (Goodacre et al., 1998). Nevertheless, there are limitations to clustering techniques as biological systems can be variable and statistical significance can be lost. When we performed the statistical Mann–Whitney test on the continuous optical densities of the isolates, UPEC was significantly different than the other *E. coli* tested in diluted TSB. However, discretization of the data occurred when the *E. coli* were sorted into the four biofilm levels, and significance was lost. Even with a loss of significance, the sorting of *E. coli* into the four classes of biofilm production is central to deliberation and reflection regarding similarities and differences among strains.

### Potential Impact of Subpathotypes and Pathogenesis on Biofilm Formation

In M63 medium, APEC and NMEC were significantly different from the other *E. coli* when their biofilm levels were categorized. The bulk of APEC and NMEC strains were classified either as negligible or low biofilm producers while UPEC, AFEC, and HFEC strains could not be differentiated. The limited biofilm production of the APEC and NMEC subpathotypes and the inability to separate *E. coli* based on their host of isolation (human or avian) leads to further evidence that APEC may be a zoonotic pathogen (Johnson et al., 2008; Tivendale et al., 2010; Nicholson et al., 2016).

Although UPEC could not be differentiated from AFEC and HFEC in M63 broth, it had statistically greater ODs in diluted TSB than the other *E. coli* tested. Undoubtedly, the ability of UPEC to produce biofilms is well-documented (Anderson et al., 2003; Soto et al., 2007). It is well-known that virulence factors associated with the adhesion of UPEC in the host include the F1C, P, S, and Type I pili as well as the Dr adhesins (Wright and Hultgren, 2006; Flores-Mireles et al., 2015). If the ability to produce biofilms promotes adherence by UPEC, the negligible or weaker biofilms produced by APEC and NMEC may be expected as expulsion from the urinary tract is not a major impediment to their ability to cause disease. Nevertheless, the UPEC selected for this study may have been inadvertently selected by the ability to survive in a urinary catheter as the isolates studied here were isolated from hospitalized patients; survival in a catheter presumably may be aided by a biofilm. Unfortunately, there is no way to ascertain whether these isolates were isolated from urine or urinary catheters.

Interestingly, both groups known to reside in the intestinal tract, AFEC (69.7%) and HFEC (82.4%), produce more biofilms in the rich medium, BHI. A different gut dwelling *E. coli* pathotype, enteroaggregative *E. coli* (EAEC) has also shown an ability to produce robust biofilms in BHI (Schiebel et al., 2017), suggesting that *E. coli* of the gut may be more suited to biofilm production in nutrient-rich conditions.

### Association Between Phylogenetic Typing and Biofilm Formation

In Clermont’s original typing scheme, *E. coli* strains were subtyped into four groups: A, B1, B2, and D (Clermont et al., 2000). Original phylogenetic group A isolates are found as commensals in the human gut (Duriez et al., 2001), in sediment environments (Vignaroli et al., 2012), or as pathogens in poultry (Logue et al., 2017). However, phylogenetic group A may be best known for containing the laboratory strain MG1655 K12 (Nash et al., 2010). When the ability to produce biofilms was examined for phylogenetic group A isolates, a majority of isolates formed negligible biofilms in each media, the only group to do so in all three media types. While group A and B1 have been proposed to be sister clades (Lecointre et al., 1998) and have been shown to be inversely related in some aquatic environments (Jang et al., 2014), group B1 produced more high-level biofilms than any other group in diluted TSB. Group B1 has been shown to occur more often than group A in aquatic environments during high temperature seasons, and the production of biofilms could promote adhesion to surfaces in these warmer temperatures (Jang et al., 2014). Phylogenetic group B2 has long been known as a group containing many human pathogens, including NMEC (Wijetunge et al., 2015; Logue et al., 2017), UPEC (Rodriguez-Siek et al., 2005a; Soto et al., 2007; Logue et al., 2017), and strains belonging to irritable bowel disease patients (Petersen et al., 2009). Phylogenetic group B2 had significantly different biofilm levels than the other groups in media that was more nutrient rich – diluted TSB and BHI (**Table 2**). In these media, B2 isolates produced more biofilms than any other group. Finally, significant differences for group D occurred solely in BHI as group D produced more moderate level biofilms than any other group. While classification of the isolates by Clermont’s original phylogenetic typing scheme yielded interesting results, the scheme was revised in 2013 as a means to better classify some of the inconsistencies in the original typing scheme. We include the scheme here as a means to interpret the potential influence of the original phylogenetic group for research already completed.

In 2008, Gordon et al. (2008) found that 80–85% of the phylogenetic group memberships assignment under Clermont’s original scheme were correct with assignment to groups B1 and B2 were correct for 95% of assignments. Therefore, the scheme was revised in 2013 to include *E. coli* specific groups that are used to classify *E. coli*: A, B1, B2, C, D, E, F, and Clade I (Clermont et al., 2013). Logue et al. (2017) found significant phylogenetic group changes when classification of ExPEC and commensal *E. coli* strains were examined using the new phylogenetic typing scheme . In APEC and UPEC, significant changes were observed from groups A to C and D to E or F (Logue et al., 2017). In NMEC, group D isolates changed to group F (Logue et al., 2017). Upon our re-categorization according to Clermont’s revised phylogenetic typing scheme (Clermont et al., 2013), we found *E. coli* isolates of group E to be the most prolific producers of biofilms in minimal medium. Unfortunately, little is known about group E, but it may consist of pathogens such as diarrheagenic *E. coli* (Bohlin et al., 2014). Unlike phylogenetic group E, group F produced the lowest optical density, suggesting poor biofilm capability in minimal and nutrient limited media under the conditions of this study. The relatively low production of biofilms by group F is thought-provoking as group F strains have been shown to contain relatively high numbers of virulence, resistance, and pathogenicity island associated genes when examined in *E. coli* stains isolated from poultry (Logue et al., 2017). The B2 phylogenetic group has been suggested to be a sister group to phylogenetic group F (Jaureguy et al., 2008; Clermont et al., 2011), but we found that tested B2 strains were relatively good at producing biofilms in all media, significantly contrasting with group F. Thus, an investigation of the genetic differences between phylogenetic group B2 and F is warranted.

## Conclusion

The work presented here is novel and uses a machine learning based approach to remove human bias in the classification of biofilms among a collection or ExPEC and their commensal counterparts. This study has also demonstrated that there was no significant difference between the *E. coli* isolates from animal and human hosts when they were not separated by commensal or subpathotype categories. When isolates were separated by their demonstrated pathogenesis in their respective host, biofilm production for APEC and NMEC was significantly different from other *E. coli* in M63 as these subpathotypes produced negligible or weaker biofilms. Interestingly, UPEC was a significantly better biofilm former than the other *E. coli* tested in diluted TSB. Clearly, further work is necessary to elucidate the mechanisms of pathogenesis and virulence factors of ExPEC that contribute to biofilm formation in moderate and high-level biofilm formers. When considering the prolific biofilm forming abilities of phylogenetic group E in M63, it is evident that more research is warranted to explore this relatively new phylogenetic group and its potential association with disease in human and animal hosts.

## Data Availability

All data from this study has been made available in the supplemental files. Isolates used in the study can be requested but may not be available due to current work that is ongoing. Isolates from our collaborators will require request directly from them. All materials will be subjected to a materials transfer agreement (MTA).

## Ethics Statement

The work presented was covered under the Institutional Biosafety Committee (IBC) approval 04-D/I-005-A/H at Iowa State University. No humans were involved in this study and the isolates used were collected from previous studies and de-identified when they were supplied to us. No animals were used in this study and isolates were collected from multiple studies dating back more than 20 years at multiple institutions.

## Author Contributions

DN carried out the research, data analysis, and drafting of the paper. JK designed and performed the statistical analysis and co-authored the paper. TC provided assistance in strain analysis and data generation. YW provided assistance in molecular analysis including phylogenetic typing and contributed to the writing of the paper. NB provided assistance in the microbial analysis, biofilm analysis, and editing of the paper. LN provided materials for the study and writing of the paper. CL helped design the study, draft the paper, and provided materials for the study.

## Conflict of Interest Statement

The authors declare that the research was conducted in the absence of any commercial or financial relationships that could be construed as a potential conflict of interest.

## References

[B1] Alkeskas A. Ogrodzki P. Saad M. Masood N. Rhoma N. R. Moore K. (2015). The molecular characterisation of *Escherichia coli* K1 isolated from neonatal nasogastric feeding tubes. 15:449. 10.1186/s12879-015-1210-7 26497222 PMC4620641

[B2] Amaral L. D. (2004). Drinking water as a risk factor to poultry health. 6 191–199. 10.1590/S1516-635X2004000400001

[B3] Anderson G. G. Palermo J. J. Schilling J. D. Roth R. Heuser J. Hultgren S. J. (2003). Intracellular bacterial biofilm-like pods in urinary tract infections. 301 105–107. 10.1126/science.1084550 12843396

[B4] Beloin C. Roux A. Ghigo J.-M. (2008). *Escherichia coli* biofilms. 322 249–289. 10.1007/978-3-540-75418-3_12 PMC2864707 18453280

[B5] Bohlin J. Brynildsrud O. B. Sekse C. Snipen L. (2014). An evolutionary analysis of genome expansion and pathogenicity in *Escherichia coli*. 15:882. 10.1186/1471-2164-15-882 25297974 PMC4200225

[B6] Clermont O. Bonacorsi S. Bingen E. (2000). Rapid and simple determination of the *Escherichia coli* phylogenetic group. 66 4555–4558. 10.1128/AEM.66.10.4555-4558.2000 11010916 PMC92342

[B7] Clermont O. Christenson J. K. Denamur E. Gordon D. M. (2013). The Clermont *Escherichia coli* phylo-typing method revisited: improvement of specificity and detection of new phylo-groups. 5 58–65. 10.1111/1758-2229.12019 23757131

[B8] Clermont O. Olier M. Hoede C. Diancourt L. Brisse S. Keroudean M. (2011). Animal and human pathogenic *Escherichia coli* strains share common genetic backgrounds. 11 654–662. 10.1016/j.meegid.2011.02.005 21324381

[B9] Davey M. E. O’toole G. A. (2000). Microbial biofilms: from ecology to molecular genetics. 64 847–867. 10.1128/MMBR.64.4.847-867.2000 PMC99016 11104821

[B10] Donlan R. M. Costerton J. W. (2002). Biofilms: survival mechanisms of clinically relevant microorganisms. 15 167–193. 10.1128/CMR.15.2.167-193.2002 11932229 PMC118068

[B11] Duriez P. Clermont O. Bonacorsi S. Bingen E. Chaventre A. Elion J. (2001). Commensal *Escherichia coli* isolates are phylogenetically distributed among geographically distinct human populations. 147 1671–1676. 10.1099/00221287-147-6-1671 11390698

[B12] Flemming H.-C. Wingender J. (2010). The biofilm matrix. 8 623–633. 10.1038/nrmicro2415 20676145

[B13] Flores-Mireles A. L. Walker J. N. Caparon M. Hultgren S. J. (2015). Urinary tract infections: epidemiology, mechanisms of infection and treatment options. 13 269–284. 10.1038/nrmicro3432 25853778 PMC4457377

[B14] Gaschignard J. Levy C. Romain O. Cohen R. Bingen E. Aujard Y. (2011). Neonatal bacterial meningitis: 444 cases in 7 years. 30 212–217. 10.1097/INF.0b013e3181fab1e7 21416693

[B15] Ghasemi-Varnamkhasti M. Mohtasebi S. S. Siadat M. Balasubramanian S. (2009). Meat quality assessment by electronic nose (machine olfaction technology). 9:6058. 10.3390/s90806058 22454572 PMC3312430

[B16] Goodacre R. Timmins É. M. Burton R. Kaderbhai N. Woodward A. M. Kell D. B. (1998). Rapid identification of urinary tract infection bacteria using hyperspectral whole-organism fingerprinting and artificial neural networks. 144 1157–1170. 10.1099/00221287-144-5-1157 9611790

[B17] Gordon D. M. Clermont O. Tolley H. Denamur E. (2008). Assigning *Escherichia coli* strains to phylogenetic groups: multi-locus sequence typing versus the PCR triplex method. 10 2484–2496. 10.1111/j.1462-2920.2008.01669.x 18518895

[B18] Hannan T. J. Totsika M. Mansfield K. J. Moore K. H. Schembri M. A. Hultgren S. J. (2012). Host–pathogen checkpoints and population bottlenecks in persistent and intracellular uropathogenic *Escherichia coli* bladder infection. 36 616–648. 10.1111/j.1574-6976.2012.00339.x 22404313 PMC3675774

[B19] Haugen J.-E. Kvaal K. (1998). Electronic nose and artificial neural network. 49 S273–S286. 10.1016/S0309-1740(98)90054-7 22060717

[B20] Jang J. Di D. Y. Lee A. Unno T. Sadowsky M. J. Hur H. G. (2014). Seasonal and genotypic changes in *Escherichia coli* phylogenetic groups in the Yeongsan River basin of South Korea. 9:e100585. 10.1371/journal.pone.0100585 24999864 PMC4085056

[B21] Jaureguy F. Landraud L. Passet V. Diancourt L. Frapy E. Guigon G. (2008). Phylogenetic and genomic diversity of human bacteremic *Escherichia coli* strains. 9:560. 10.1186/1471-2164-9-560 19036134 PMC2639426

[B22] Johnson T. J. Kariyawasam S. Wannemuehler Y. Mangiamele P. Johnson S. J. Doetkott C. (2007). The genome sequence of avian pathogenic *Escherichia coli* strain O1:K1:H7 shares strong similarities with human extraintestinal pathogenic *E. coli* genomes. 189 3228–3236. 17293413 10.1128/JB.01726-06 PMC1855855

[B23] Johnson T. J. Logue C. M. Wannemuehler Y. Kariyawasam S. Doetkott C. Debroy C. (2009). Examination of the source and extended virulence genotypes of *Escherichia coli* contaminating retail poultry meat. 6 657–667. 10.1089/fpd.2009.0266 19580453 PMC3145168

[B24] Johnson T. J. Siek K. E. Johnson S. J. Nolan L. K. (2005). DNA sequence and comparative genomics of pAPEC-O2-R, an avian pathogenic *Escherichia coli* transmissible R plasmid. 49 4681–4688. 10.1128/AAC.49.11.4681-4688.2005 16251312 PMC1280136

[B25] Johnson T. J. Wannemuehler Y. Johnson S. J. Stell A. L. Doetkott C. Johnson J. R. (2008). Comparison of extraintestinal pathogenic *Escherichia coli* strains from human and avian sources reveals a mixed subset representing potential zoonotic pathogens. 74 7043–7050. 10.1128/AEM.01395-08 18820066 PMC2583479

[B26] Kruskal W. H. Wallis W. A. (1952). Use of ranks in one-criterion variance analysis. 47 583–621. 10.1080/01621459.1952.10483441

[B27] Lecointre G. Rachdi L. Darlu P. Denamur E. (1998). *Escherichia coli* molecular phylogeny using the incongruence length difference test. 15 1685–1695. 10.1093/oxfordjournals.molbev.a025895 9866203

[B28] Lindsay D. Geornaras I. Von Holy A. (1996). Biofilms associated with poultry processing equipment. 86 105–116. 8858863

[B29] Logue C. M. Doetkott C. Mangiamele P. Wannemuehler Y. M. Johnson T. J. Tivendale K. A. (2012). Genotypic and phenotypic traits that distinguish neonatal meningitis-associated *Escherichia coli* from fecal *E. coli* isolates of healthy human hosts. 78 5824–5830. 10.1128/AEM.07869-11 22706051 PMC3406136

[B30] Logue C. M. Wannemuehler Y. Nicholson B. A. Doetkott C. Barbieri N. L. Nolan L. K. (2017). Comparative analysis of phylogenetic assignment of human and avian ExPEC and fecal commensal *Escherichia coli* using the (previous and revised) Clermont phylogenetic typing methods and its impact on avian pathogenic *Escherichia coli* (APEC) classification. 8:283 10.3389/fmicb.2017.00283 PMC5322314 28280491

[B31] MacKay D. J. C. (2003). *Information Theory, Inference, and Learning Algorithms*. New York, NY: Cambridge University Press.

[B32] Mann H. B. Whitney D. R. (1947). On a test of whether one of two random variables is stochastically larger than the other. 18 50–60. 10.1214/aoms/1177730491

[B33] Mellata M. Johnson J. R. Curtiss R. III (2017). *Escherichia coli* isolates from commercial chicken meat and eggs cause sepsis, meningitis and urinary tract infection in rodent models of human infections. 65 103–113. 10.1111/zph.12376 28703468

[B34] Merritt J. H. Kadouri D. E. O’toole G. A. (2005). Growing and analyzing static biofilms. 1B.1.1–1B.1.17. 10.1002/9780471729259.mc01b01s00 18770545 PMC4568995

[B35] Mitchell N. M. Johnson J. R. Johnston B. Curtiss R. III Mellata M. (2015). Zoonotic potential of *Escherichia coli* isolates from retail chicken meat products and eggs. 81 1177–1187. 10.1128/AEM.03524-14 25480753 PMC4292506

[B36] Nash J. H. Villegas A. Kropinski A. M. Aguilar-Valenzuela R. Konczy P. Mascarenhas M. (2010). Genome sequence of adherent-invasive *Escherichia coli* and comparative genomic analysis with other *E. coli* pathotypes. 11:667. 10.1186/1471-2164-11-667 21108814 PMC3091784

[B37] Nicholson B. A. West A. C. Mangiamele P. Barbieri N. Wannemuehler Y. Nolan L. K. (2016). Genetic characterization of ExPEC-like virulence plasmids among a subset of NMEC. 11:e0147757. 10.1371/journal.pone.0147757 26800268 PMC4723317

[B38] Nolan L. K. Barnes H. J. Vaillancourt J.-P. Abdul-Aziz T. Logue C. M. (2013). “Colibacillosis,” in 13th Edn, eds Swayne D. E. Glisson J. R. McDougald L. R. Nolan L. K. Suarezand D. L. Naireds V. (Ames, IA: John Wiley & Sons, Ltd.) 751–805.

[B39] O’Toole G. A. (2011). Microtiter dish biofilm formation assay. 47:2437. 10.3791/2437 21307833 PMC3182663

[B40] O’Toole G. A. Pratt L. A. Watnick P. I. Newman D. K. Weaver V. B. Kolter R. (1999). “[6] Genetic approaches to study of biofilms,” in ed. Ron J. D. (Waltham, MA: Academic Press) 91–109. 10.1016/s0076-6879(99)10008-9 10547784

[B41] Peeters E. Nelis H. J. Coenye T. (2008). Comparison of multiple methods for quantification of microbial biofilms grown in microtiter plates. 72 157–165. 10.1016/j.mimet.2007.11.010 18155789

[B42] Petersen A. M. Nielsen E. M. Litrup E. Brynskov J. Mirsepasi H. Krogfelt K. A. (2009). A phylogenetic group of *Escherichia coli* associated with active left-sided inflammatory bowel disease. 9:171. 10.1186/1471-2180-9-171 19695087 PMC2736970

[B43] Picard B. Garcia J. S. Gouriou S. Duriez P. Brahimi N. Bingen E. (1999). The link between phylogeny and virulence in *Escherichia coli* extraintestinal infection. 67 546–553. 9916057 10.1128/iai.67.2.546-553.1999 PMC96353

[B44] Potera C. (1999). Forging a link between biofilms and disease. 283 1837–1839. 10.1126/science.283.5409.1837 10206887

[B45] Rodriguez-Siek K. E. Giddings C. W. Doetkott C. Johnson T. J. Fakhr M. K. Nolan L. K. (2005a). Comparison of *Escherichia coli* isolates implicated in human urinary tract infection and avian colibacillosis. 151 2097–2110. 15942016 10.1099/mic.0.27499-0

[B46] Rodriguez-Siek K. E. Giddings C. W. Doetkott C. Johnson T. J. Nolan L. K. (2005b). Characterizing the APEC pathotype. 36 241–256. 15720976 10.1051/vetres:2004057

[B47] Schiebel J. Bohm A. Nitschke J. Burdukiewicz M. Weinreich J. Ali A. (2017). Genotypic and phenotypic characteristics in association with biofilm formation in different pathotypes of human clinical *Escherichia coli* isolates of different pathotypes. 83:e01660-17. 10.1128/AEM.01660-17 28986371 PMC5717203

[B48] Schwartz D. J. Chen S. L. Hultgren S. J. Seed P. C. (2011). Population dynamics and niche distribution of uropathogenic *Escherichia coli* during acute and chronic urinary tract infection. 79 4250–4259. 10.1128/IAI.05339-11 21807904 PMC3187256

[B49] Skyberg J. A. Siek K. E. Doetkott C. Nolan L. K. (2007). Biofilm formation by avian *Escherichia coli* in relation to media, source and phylogeny. 102 548–554. 10.1111/j.1365-2672.2006.03076.x 17241361

[B50] Soto S. M. Smithson A. Martinez J. A. Horcajada J. P. Mensa J. Vila J. (2007). Biofilm formation in uropathogenic *Escherichia coli* strains: relationship with prostatitis, urovirulence factors and antimicrobial resistance. 177 365–368. 10.1016/j.juro.2006.08.081 17162092

[B51] Stepanović S. Ćirković I. Ranin L. Svabić-Vlahović M. (2004). Biofilm formation by *Salmonella* spp. and *Listeria monocytogenes* on plastic surface. 38 428–432. 10.1111/j.1472-765X.2004.01513.x 15059216

[B52] Stoll B. J. Hansen N. I. Sanchez P. J. Faix R. G. Poindexter B. B. Van Meurs K. P. (2011). Early onset neonatal sepsis: the burden of group B Streptococcal and *E. coli* disease continues. 127 817–826. 10.1542/peds.2010-2217 21518717 PMC3081183

[B53] Sturgill G. Toutain C. M. Komperda J. O’toole G. A. Rather P. N. (2004). Role of CysE in production of an extracellular signaling molecule in *Providencia stuartii* and *Escherichia coli*: loss of cysE enhances biofilm formation in *Escherichia coli*. 186 7610–7617. 10.1128/JB.186.22.7610-7617.2004 15516574 PMC524891

[B54] Tivendale K. A. Logue C. M. Kariyawasam S. Jordan D. Hussein A. Li G. (2010). Avian-pathogenic *Escherichia coli* strains are similar to neonatal meningitis *E. coli* strains and are able to cause meningitis in the rat model of human disease. 78 3412–3419. 10.1128/IAI.00347-10 20515929 PMC2916289

[B55] Tukey J. W. (1949). Comparing individual means in the analysis of variance. 5 99–114. 10.2307/3001913 18151955

[B56] Vignaroli C. Luna G. M. Rinaldi C. Di Cesare A. Danovaro R. Biavasco F. (2012). New sequence types and multidrug resistance among pathogenic *Escherichia coli* isolates from coastal marine sediments. 78 3916–3922. 10.1128/AEM.07820-11 22447595 PMC3346399

[B57] Wijetunge D. S. S. Gongati S. Debroy C. Kim K. S. Couraud P. O. Romero I. A. (2015). Characterizing the pathotype of neonatal meningitis causing *Escherichia coli* (NMEC). 15:211. 10.1186/s12866-015-0547-9 26467858 PMC4606507

[B58] Wright K. J. Hultgren S. J. (2006). Sticky fibers and uropathogenesis: bacterial adhesins in the urinary tract. 1 75–87. 10.2217/17460913.1.1.75 17661687

