# Supplementary material for: The Impact of Media, Phylogenetic Classification, and E. coli Pathotypes on Biofilm Formation in Extraintestinal and Commensal E. coli From Humans and Animals
Source: Front Microbiol. 2018 May 8;9:902. doi: 10.3389/fmicb.2018.00902 (PMC5951942; doi:10.3389/fmicb.2018.00902)
Supplement: Supplementary file 1 [file Data_Sheet_1.docx]

**Supplementary Figure 1: Optical Densities of *E. coli* Strains by Media Type**

Biofilm formation relative to the media type with the mean OD_600_ plotted; error bars represent ± standard error of mean.

**Supplementary Figure 2: Optical densities of *E. coli* classified from their host source**

Optical densities of *E. coli* classified from their source of isolation, human (blue) or avian (green), with the three different media types.
