# Supplementary figures and images for: The Impact of Media, Phylogenetic Classification, and E. coli Pathotypes on Biofilm Formation in Extraintestinal and Commensal E. coli From Humans and Animals
Source: Front Microbiol. 2018 May 8;9:902. doi: 10.3389/fmicb.2018.00902 (PMC5951942; doi:10.3389/fmicb.2018.00902)

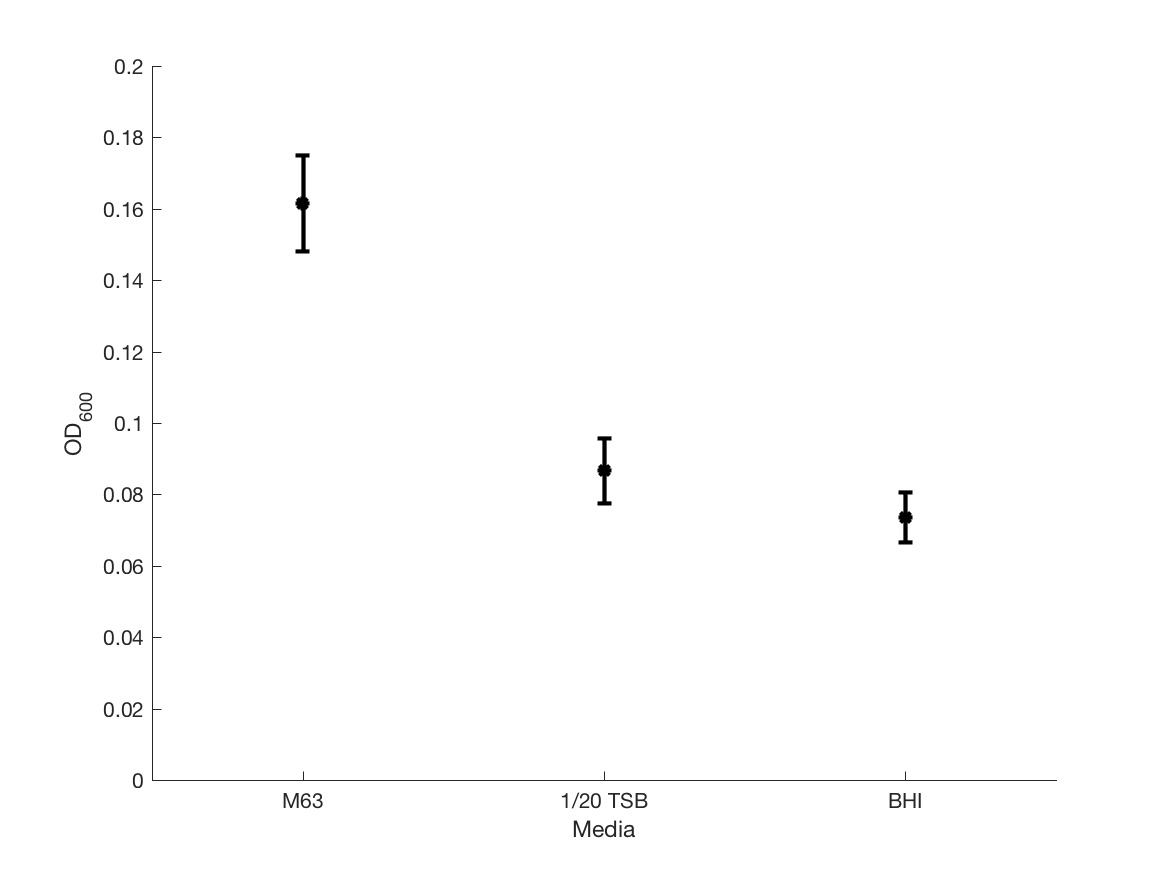

Supplement: Supplementary file 2 [file Image_1.JPEG]

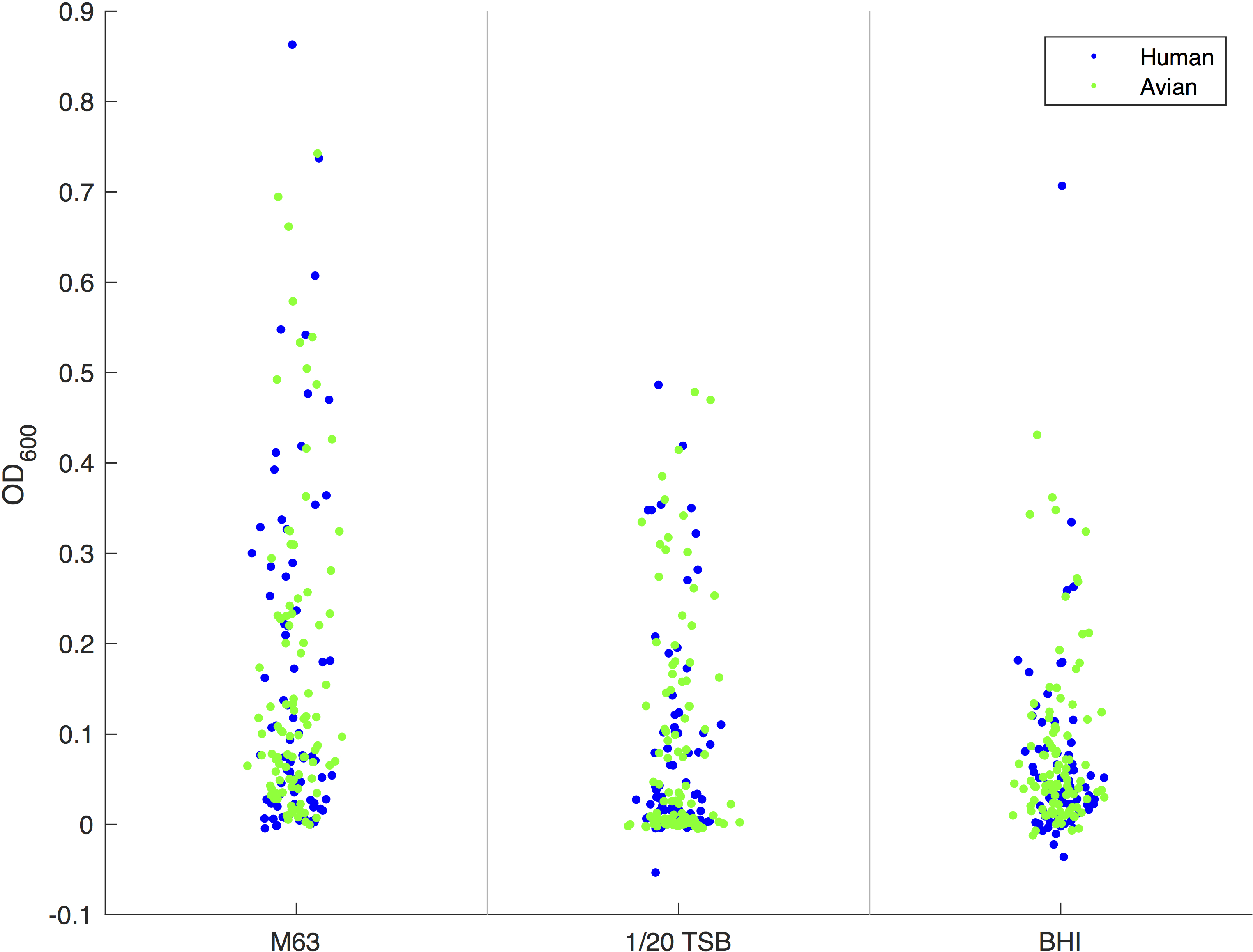

Supplement: Supplementary file 3 [file Image_2.JPEG]
