# Supplementary material for: The Impact of Media, Phylogenetic Classification, and E. coli Pathotypes on Biofilm Formation in Extraintestinal and Commensal E. coli From Humans and Animals
Source: Front Microbiol. 2018 May 8;9:902. doi: 10.3389/fmicb.2018.00902 (PMC5951942; doi:10.3389/fmicb.2018.00902)
Supplement: Supplementary file 4 [file Table_1.docx]

**Supplementary Table 1: Chi-square Discrete Categorization of *E. coli* by Commensal and Subpathotype Groups***

|  | **M63** | | | |  |
| --- | --- | --- | --- | --- | --- |
|  | **Negligible** | **Low** | **Moderate** | **High** | **p** |
| **APEC** | 56.8% | 13.6% | 20.5% | 9.1% | **0.0064** |
| **NMEC** | 31.3% | 53.1% | 6.3% | 9.4% | **0.0093** |
| **UPEC** | 28.1% | 34.4% | 28.1% | 9.4% | 0.6007 |
| **AFEC** | 27.3% | 33.3% | 24.2% | 15.2% | 0.6371 |
| **HFEC** | 32.4% | 23.5% | 29.4% | 14.7% | 0.4956 |
|  |  |  |  |  |  |
|  | **TSB** | | | |  |
|  | **Negligible** | **Low** | **Moderate** | **High** | **p** |
| **APEC** | 65.9% | 18.2% | 6.8% | 9.1% | 0.5807 |
| **NMEC** | 53.1% | 18.8% | 21.9% | 6.3% | 0.1619 |
| **UPEC** | 43.8% | 15.6% | 15.6% | 25.0% | 0.0505 |
| **AFEC** | 57.6% | 21.2% | 6.1% | 15.2% | 0.6197 |
| **HFEC** | 73.5% | 11.8% | 8.8% | 5.9% | 0.2969 |
|  |  |  |  |  |  |
|  | **BHI** | | | |  |
|  | **Negligible** | **Low** | **Moderate** | **High** | **p** |
| **APEC** | 38.6% | 36.4% | 20.5% | 4.5% | 0.6064 |
| **NMEC** | 37.5% | 34.4% | 21.9% | 6.3% | 0.7880 |
| **UPEC** | 31.3% | 40.6% | 25.0% | 3.1% | 0.7300 |
| **AFEC** | 30.3% | 54.5% | 9.1% | 6.1% | 0.2530 |
| **HFEC** | 17.6% | 44.1% | 23.5% | 14.7% | 0.0835 |

* Percentages may not add up to 100.0% due to rounding.
Values in bold are significant to α < 0.05
