# Supplementary material for: The Impact of Media, Phylogenetic Classification, and E. coli Pathotypes on Biofilm Formation in Extraintestinal and Commensal E. coli From Humans and Animals
Source: Front Microbiol. 2018 May 8;9:902. doi: 10.3389/fmicb.2018.00902 (PMC5951942; doi:10.3389/fmicb.2018.00902)
Supplement: Supplementary file 5 [file Table_2.docx]

**Supplementary Table 2: Chi-square Discrete Categorization of *E. coli* by Clermont’s Revised Phylogenetic Groups***

|  | **M63** | | | |  |
| --- | --- | --- | --- | --- | --- |
|  | **Negligible** | **Low** | **Moderate** | **High** | **p** |
| **A** | 60.0% | 16.0% | 8.0% | 16.0% | **0.0244** |
| **B1** | 18.2% | 40.9% | 36.4% | 4.5% | 0.0787 |
| **B2** | 18.4% | 39.5% | 26.3% | 15.8% | 0.0717 |
| **C** | 57.1% | 19.0% | 19.0% | 4.8% | 0.1900 |
| **D** | 20.8% | 50.0% | 20.8% | 8.3% | 0.1274 |
| **E** | 11.8% | 29.4% | 29.4% | 29.4% | **0.0291** |
| **F** | 67.9% | 14.3% | 14.3% | 3.6% | **0.0024** |
|  |  |  |  |  |  |
|  | **1/20 TSB** | | | |  |
|  | **Negligible** | **Low** | **Moderate** | **High** | **p** |
| **A** | 88.0% | 8.0% | 0.0% | 4.0% | **0.0161** |
| **B1** | 45.5% | 9.1% | 13.6% | 31.8% | **0.0176** |
| **B2** | 42.1% | 26.3% | 23.7% | 7.9% | **0.0078** |
| **C** | 66.7% | 19.0% | 14.3% | 0.0% | 0.3483 |
| **D** | 50.0% | 20.8% | 8.3% | 20.8% | 0.4438 |
| **E** | 47.1% | 29.4% | 11.8% | 11.8% | 0.5437 |
| **F** | 78.6% | 7.1% | 3.6% | 10.7% | 0.1193 |
|  |  |  |  |  |  |
|  | **BHI** | | | |  |
|  | **Negligible** | **Low** | **Moderate** | **High** | **p** |
| **A** | 56.0% | 16.0% | 8.0% | 20.0% | **0.0002** |
| **B1** | 36.4% | 31.8% | 22.7% | 9.1% | 0.7861 |
| **B2** | 18.4% | 63.2% | 13.2% | 5.3% | **0.0258** |
| **C** | 42.9% | 38.1% | 14.3% | 4.8% | 0.6573 |
| **D** | 25.0% | 33.3% | 37.5% | 4.2% | 0.1456 |
| **E** | 17.6% | 47.1% | 29.4% | 5.9% | 0.5465 |
| **F** | 28.6% | 50.0% | 21.4% | 0.0% | 0.3986 |

* Percentages may not add up to 100.0% due to rounding.

Values in bold are significant to α < 0.05
